# Dunglison’s Medical Dictionary

**Published:** 1874-02

**Authors:** 


A Dictionary of Medical Science, *with the Accentuation and Etymology of the terms, and the French and other Synonyms. By Robley Dunglison, M.D. L.L.D., etc. A new edition, enlarged and thoroughly revised by Richard J. Dunglison, M. D., Philadelphia. Henry C. Lea,* 1874—*Octavo, pp.* 1131. *Cloth,* $5.60; *Leather,* $7.50.

We have been accustomed to take up Dunglison's Medical Dictionary with much of the feeling of deference and deep respect with which we ever regarded its dignified and erudite author. With a history of forty years of unexampled success and universal indorsement by the medical profession of the western continent, it would be presumption in any living medical American to essay its review. No reviewer, however able, can add to its fame; no captious critic, however caustic, can remove a single stone from its firm and enduring foundation. It is destined, as a colossal monument, to perpetuate the solid and richly deserved fame of Robley Dunglison to coming generations.

In looking to the labors of the editor and of the publisher, we observe that the work has been greatly enlarged, both by the addition of more than six thousand terms, and one hundred pages to the volume, as well as a moderate expansion of both the length and the breadth of the page. In this connection we would modestly remark, that we regard the very general *uniformity* in the size of the page, to be found throughout the great mass of the medical works published by Lea, as a commendable feature, and one which should not be lost, except for good and sufficient reasons.

The large additions made to the vocabulary, we think, will be welcomed by the profession as supplying the want of a lexicon fully up with the march of science, which has been increasingly felt for some years past. The accentuation of terms is very complete, and, as far as we have been able to examine it, very excellent. We hope it may be the means of securing greater uniformity of pronunciation among medical men.

In a cursory search for the new additions and improvements introduced by the editor, our eye falls upon "Darby's Fluid," and following the reference to "Condy's Fluid," which "is supposed to be a concentrated solution of the permanganate of potassa." We learn "it is a good anti-bromic. Darby's Fluid is a similar preparation." Darby's Fluid, which originated in the South, is generally understood to be what the Dictionary calls an *arcanum*, and is believed to be a weak solution of chlorinated soda, tinted with the permanganate. As we do not find the term Jayne's Expectorant in the list, we infer that Darby's Fluid has crept in by mistake.

The work is well gotten up and substantially bound, as it ought to be, and fitly takes its place beside Webster's great quarto. We cheerfully donate our old copy of Dunglison to the Society for the Diffusion of Useful Knowledge, and take the new one upon our table for our daily companion.

